# *Bacillus coagulans: *a viable adjunct therapy for relieving symptoms of rheumatoid arthritis according to a randomized, controlled trial

**DOI:** 10.1186/1472-6882-10-1

**Published:** 2010-01-12

**Authors:** David R Mandel, Katy Eichas, Judith Holmes

**Affiliations:** 1 David R. Mandel, MD, Inc., Mayfield Village, OH 44143, USA; 2 David R. Mandel, MD, Inc., Chardon, OH 44024, USA

## Abstract

**Background:**

Lactic acid-producing bacteria (LAB) probiotics demonstrate immunomodulating and anti-inflammatory effects and the ability to lessen the symptoms of arthritis in both animals and humans. This randomized, double-blind, placebo-controlled, parallel-design, clinical pilot trial was conducted to evaluate the effects of the LAB probiotic preparation, *Bacillus coagulans *GBI-30, 6086, on symptoms and measures of functional capacity in patients with rheumatoid arthritis (RA) in combination with pharmacological anti-arthritic medications.

**Methods:**

Forty-five adult men and women with symptoms of RA were randomly assigned to receive *Bacillus coagulans *GBI-30, 6086 or placebo once a day in a double-blind fashion for 60 days in addition to their standard anti-arthritic medications. Arthritis activity was evaluated by clinical examination, the American College of Rheumatology (ACR) criteria, the Stanford Health Assessment Questionnaire Disability Index (HAQ-DI), and laboratory tests for erythrocyte sedimentation rate (ESR) and C-reactive protein (CRP).

**Results:**

Subjects who received *Bacillus coagulans *GBI-30, 6086 experienced borderline statistically significant improvement in the Patient Pain Assessment score (*P *= .052) and statistically significant improvement in Pain Scale (*P *= .046) vs placebo. Compared with placebo, *Bacillus coagulans *GBI-30, 6086 treatment resulted in greater improvement in patient global assessment and self-assessed disability; reduction in CRP; as well as the ability to walk 2 miles, reach, and participate in daily activities. There were no treatment-related adverse events reported throughout this study.

**Conclusions:**

Results of this pilot study suggest that adjunctive treatment with *Bacillus coagulans *GBI-30, 6086 LAB probiotic appeared to be a safe and effective for patients suffering from RA. Because of the low study population size, larger trials are needed to verify these results.

**Trial registration:**

ACTRN12609000435280

## Background

Probiotics have been used to treat and prevent a wide range of infectious and inflammatory diseases [1,2]. Of particular interest are lactic acid bacteria (LAB) probiotics and their immunomodulating and anti-inflammatory effects, which have been shown to lessen the symptoms of arthritis [3-8].

Rheumatoid arthritis (RA) affects more than 1.3 million American adults [9]. It commonly leads to significant disability and compromises quality of life. Pharmacological treatments for arthritis target the inflammatory process by suppressing the host reaction. Despite the number of effective pharmacological agents available today, a substantial proportion of patients will experience persistent, low-level disease activity [10]. This underscores the need for adjunctive therapies that are safe and can help relieve the painful symptoms of arthritis.

RA is an autoimmune disorder in which unchecked immune and inflammatory responses cause articular pain and eventually cartilage degradation and bone destruction [11]. Disease develops when there is an imbalance in the cytokine network, either from excess production of pro-inflammatory cytokines or from inadequate natural anti-inflammatory mechanisms [12]. Evidence has shown that there is a relationship between the gastrointestinal microbiota, the mucosal and systemic immune responses, and the development of arthritis [5,13,14]. LAB have been shown to significantly downregulate proinflammatory cytokines (eg, IFN-γ, IL-12, TNF-α) without altering regulatory cytokines (eg, IL-10, TGF-β) to cause anti-inflammatory effects that alleviate RA symptoms [3,14-19]. Therefore, it might be speculated that therapeutic strategies that aim to normalize the gut microflora in order to maintain proper gastrointestinal and immune system function may downregulate the abnormal inflammatory response and alleviate symptoms of RA.

GanedenBC^30^*(Bacillus coagulans *GBI-30, 6086, Ganeden Biotech, Inc., Mayfield Heights, OH) is a strain of LAB that can withstand the low pH of stomach acid is activated in the intestines to modulate the gut microflora and the immune response [18-20]. The objective of this pilot study was to evaluate the effects of *Bacillus coagulans *GBI-30, 6086 on RA symptoms and the functional ability of patients with RA when used in combination with pharmacological anti-rheumatic medications.

## Methods

### Study design

This was a randomized, double-blind, placebo-controlled, parallel-design study of *Bacillus coagulans *GBI-30, 6086 LAB probiotic as an adjunctive therapy for the relief of symptoms of RA. The objective was to evaluate the effects of *Bacillus coagulans *GBI-30, 6086 on RA symptoms compared with placebo.

The treatment allocation scheme was generated and assigned by a third party who did not have direct patient contact. The treatment assignments were in sealed, tamper-proof, blinded envelopes and were handed out in sequential fashion according to a computer-generated randomization list. Study personnel did not have access to the randomization scheme or blinding process. Collected data were concealed in a pouch that remained unopened until all results were submitted.

This study was registered with the Australian New Zealand Clinical Trials Registry.

### Patient population

Forty-five adult men and women with symptoms of RA were included in the study. Sample size was determined by power analysis. Subjects were recruited by the primary investigator from his daily practice and follow-up visits were conducted at the practice.

Inclusion criteria included men and women with RA for at least 1 year and up to 80 years of age. The criteria for the diagnosis of RA included four or more of the following symptoms: 1) morning stiffness lasting at least 1 hour; 2) soft tissue swelling in 3 or more joint areas observed by a physician; 3) swelling of the proximal interphalangeal, metacarpophalangeal, or wrist joints; 4) symmetric swelling; 5) rheumatoid nodules; 6) the presence of rheumatoid factor; and 7) radiographic erosions and/or periarticular osteopenia in hand and/or wrist joints. The first 4 criteria must have been present for at least 6 weeks for inclusion in the study.

The exclusion criteria were pregnancy, chronic inflammatory bowel disease, kidney disease, liver disease, exposure to > 10 mg/day of prednisolone, or treatment with other probiotic products.

Patients read and signed an approved consent form prior to enrollment in the study. The study protocol and test product(s) information were approved by an Institutional Review Board (IRB; Schulman Associates, Cincinnati, OH) prior to the beginning of the study.

### Study treatment

Patients in each group underwent a baseline physical assessment and then were randomized in a double-blind manner to receive either placebo or 1 caplet of *Bacillus coagulans *GBI-30, 6086 (2 billion CFU) daily at approximately the same time each day, regardless of meals, for a period of 60 days. The probiotic preparation includes *Bacillus coagulans *GBI-30, 6086, green tea extract, methylsulfonylmethane, and vitamins and minerals (including vitamins A, B, C, D, E, folic acid, and selenium). The matching placebo contained microcrystalline cellulose. Adherence to the study protocol was determined by caplet count at each examination.

### Patient assessment

The 45 men and women enrolled in the study were randomly assigned to receive *Bacillus coagulans *GBI-30, 6086 or placebo once a day for 60 days. Patients were examined at the primary investigator's practice at baseline, at 30 days, and at 60 days to assess symptoms and measures of disease activity. Blood draws were performed at each visit. The primary outcome was change from baseline compared with the end of the study period obtained by the American College of Rheumatology (ACR) [21] criteria questionnaire and the Stanford Health Assessment Questionnaire Disability Index (HAQ-DI) [22]. Outcomes were classified as "global" (sensitive to change in clinical trials) and "individual" (relatively insensitive to change in clinical trials). The global outcomes included patients' global assessment, pain assessment, and disability assessment, as well as physicians' global assessment, assessment of total painful joints out of 68 joints assessed, total swollen joints out of 66 joints assessed, and erythrocyte sedimentation rate (ESR) and C-reactive protein (CRP) levels from ACR and pain from the HAQ-DI. Individual outcomes were assessed based on hygiene (ability to wash and dry the body, take a tub bath, get on and off the toilet) and the ability to dress and groom, arise, eat, walk 2 miles, reach, grip, and participate in daily activities. Serious adverse events were reported directly to the sponsor and IRB by each investigator.

### Statistical analysis

For each outcome, summary measures based on the change from baseline to 60 days were obtained for each group and group differences were tested. For global variables, each response was normalized (using Blom scores) and the difference (end minus baseline) was obtained to provide a change score. A Student's *t *test was performed to test for a difference in the mean change score between subjects randomized to *Bacillus coagulans *GBI-30, 6086 vs placebo. The effectiveness of the normalization was checked via the Shapiro-Wilk test for normality and the folded form F statistic to test for equality of group variances. For individual outcomes, due to their discrete nature, we utilized the binary indicator of a difference score of less than zero, indicating some improvement in the score for the item. Barnard's exact unconditional test was used to compare the proportions who improved in the *Bacillus coagulans *GBI-30, 6086 group vs placebo for each individual scale. Two-sided *P *< .05 was considered statistically significant. Ninety-five percent confidence intervals were computed for the group difference for each outcome. For continuous outcomes, confidence intervals for mean differences were obtained based on a *t *test for normalized scores. For binary outcomes, exact confidence intervals for the difference in proportions were computed.

Analyses were performed using SAS Version 8.0 (SAS System, SAS Institute, Cary, NC) and StatXact Version 4.0 (Cytel Software Corporation, Cambridge, MA).

## Results

**Figure 1:**
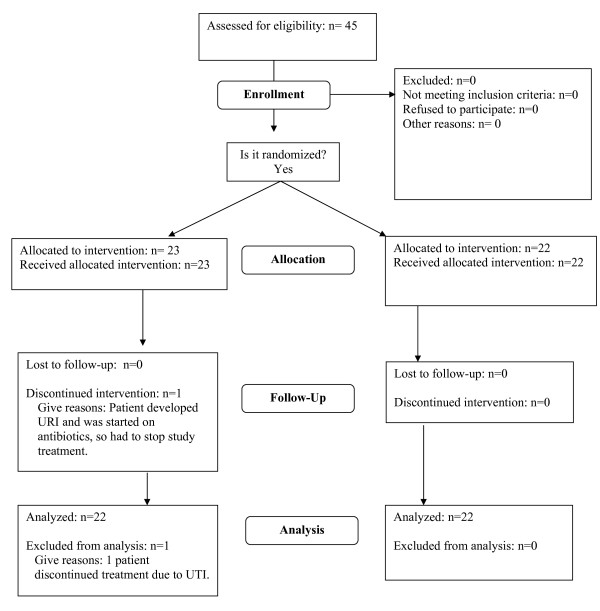
**Study flowchart**.

Forty-five men and women who had RA for at least 1 year were enrolled in the study and randomly assigned to receive *Bacillus coagulans *GBI-30, 6086 or placebo once a day for 60 days (Figure 1). The majority of subjects were female (81.8%) and all were Caucasian. Subjects included in the study were between 36 and 82 years of age. The average age was 62.5 years. Although subjects over the age of 80 were excluded from the study because many subjects past this age have multiple health issues that might impact their participation or end results, 2 patients over the age of 80 were included because they met all other study criteria and were in good overall health. The subjects' standard of care for RA was not altered during the course of the study. Most subjects were taking and continued to take disease modifying antirheumatic drugs (DMARDs) (17 in the placebo group and 18 in the study treatment group) and 5 subjects continued to take non-steroidal anti-inflammatory drugs (NSAIDs) (3 in the placebo group and 2 in the study treatment group). Four subjects were not taking medication for RA (3 in the placebo group and 1 in the treatment group). One subject developed an upper respiratory infection (URI) and was started on antibiotics, so the study treatment was discontinued. This subject did not return for further visits and was, therefore, excluded from analyses. Although this was not an intent-to-treat analysis due to the 1 subject who discontinued treatment, each study group included 22 patients for analysis.

### Efficacy

Subjects who received *Bacillus coagulans *GBI-30, 6086 experienced borderline statistically significant improvement from baseline in the Patient Pain Assessment score (*P *= .052) and statistically significant improvement from baseline in the Pain Scale (*P *= .046) compared with subjects randomized to receive placebo. *Bacillus coagulans *GBI-30, 6086 treatment resulted in greater improvement in patient global assessment, patient self-assessed disability, and reduction in total CRP (Table 1). There were no significant differences in the physician global assessment or physician assessment of painful and swollen joints. For the HAQ individual disability scores, the ability to walk 2 miles was marginally significant (*P *= .072) and the ability to reach was not quite significant (*P *= .11) (Table 2). *Bacillus coagulans *GBI-30, 6086 also outperformed placebo for improvement in the ability to participate in daily activities.

**Table 1:** Mean change in global outcomes from baseline to 60 days

	Placebo/*Bacillus coagulans *GBI-30, 6086	Difference in Means (95% CI)	*P *Value^a^
Score	22/22	0.006 (-0.33, 0.35)	.97

Pain	21/22	0.46 (0.01, 0.91)	.046

Patient global	22/22	0.047 (-0.38, 0.47)	.83

Patient pain	22/22	0.41 (-0.004, 0.82)	.052

Patient disability	22/22	0.19 (-0.16, 0.54)	.28

Physician global	22/22	0.019 (-0.62, 0.66)	.95

Painful joints	22/22	-0.074 (-0.81, 0.66)	.84

Swollen joints	22/22	0.011 (-0.62, 0.64)	.97

ESR	21/21	-0.054 (-0.49, 0.38)	.80

CRP	22/22	0.008 (-0.52, 0.53)	.98

Score = the mean of the eight category scores in the HAQ.

ESR = erythrocyte sedimentation rate.

CRP = C-reactive protein.

^a^*P *values calculated using Student's *t *test for group differences in the mean change scores from baseline to 60 days (95% confidence interval).

**Table 2:** Proportion of patients who improved in individual outcomes from baseline to 60 days

	Placebo		*Bacillus coagulans *GBI-30, 6086			
			
	Number Improved/n	Proportion	Number Improved/n	Proportion	Difference in Proportions (95% CI)	*P *Value^a^
Arising	3/22	0.14	4/21	0.19	-0.054 (-0.36, 0.24)	.71

Walking 2 miles	2/22	0.091	7/22	0.32	-0.23 (-0.51, 0.09)	.072

Daily activities	2/22	0.091	4/22	0.18	-0.091 (-0.38, 0.21)	.53

Dressing and grooming	4/22	0.18	4/22	0.18	0 (-0.30, 0.30)	1.0

Eating	6/22	0.27	4/22	0.18	0.09 (-0.22, 0.39)	.53

Hygiene	2/22	0.091	2/22	0.091	0 (-0.28, 0.28)	1.0

Reach	4/22	0.18	9/22	0.41	-0.23 (-0.52, 0.09)	.11

Grip	5/22	0.23	4/22	0.18	0.045 (-0.26, 0.35)	.81

^a^*P *values calculated using Barnard's exact unconditional test to compare the proportion who improved in both groups for each individual scale.

Eight subjects in the *Bacillus coagulans *GBI-30, 6086 group met ACR 20 criteria vs 6 subjects in the placebo group. See Table 3 for an analysis of each group.

**Table 3:** Outcomes meeting ACR 20 criteria by treatment group

	*Bacillus coagulans *GBI-30, 6086		Placebo	
	
	% Change	Met ACR 20 Criteria?	% Change	Met ACR 20 Criteria?
Total painful joints	-29.9	Yes	-29.3	Yes

Total swollen joints	-17.0	No	-19.3	No

Patient global	-11.7	No	-1.5	No

Patient pain	-19.8	No	-1.6	No

Patient disability	-22.5	Yes	-2.2	No

Physician global	-21.6	Yes	-22.3	Yes

CRP/ESR	-46.0/+6.5	Yes	+4.8/+9.1	No

ACR = American College of Rheumatology.

ESR = erythrocyte sedimentation rate.

CRP = C-reactive protein.

### Safety

There were no serious adverse reactions reported throughout this study. The treatment group reported 4 adverse events including shingles, poison ivy, a cold, and leg edema, all of which were deemed unrelated to the study treatment. The placebo group reported 3 adverse events including gastrointestinal reflux, URI, and urinary tract infection (UTI).

## Discussion

The potential role of the intestinal microflora in modulating immune responses has led to an interest in using probiotics as preventive and therapeutic interventions. For example, it has been shown that the enteric microflora impact intestinal inflammatory responses and may contribute to the articular inflammatory characteristics of arthritis [13].

Rheumatoid arthritis is an autoimmune disease characterized by a loss of tolerance to autoantigens (self antigens), which triggers an inflammatory immune response that causes joint damage and functional impairment [23]. It has been speculated that infection by microbial pathogens may trigger autoimmune reactions through cross-reactivity, as environmental pathogens present antigens that mimic autoantigens [23]. Changes to the normal gastrointestinal microflora and dysregulation of the mucosal immune response to these pathogens may contribute to the development of autoimmune diseases such as RA [23]. This possibility has led investigators to evaluate the efficacy of probiotics for alleviating RA symptoms through modulation of the aberrant inflammatory autoimmune response.

Several preclinical studies have evaluated the effects of various strains of LAB probiotics on symptoms and clinical markers of arthritis. In one study [3], there was a statistically significant decrease in inflammation over 1 month in rats fed yogurt containing *Lactobacillus *GG (LGG) compared with rats fed plain yogurt or milk (*P *< .05). An earlier study [18] found that *Lactobacillus casei *reduced the incidence and development of collagen-induced arthritis in mice and downregulated the cellular and humoral immune responses to collagen in a dose-dependent manner. More recently, a study in rats with collagen-induced arthritis demonstrated that oral administration of *L casei *for 12 weeks reduced signs of arthritis, lymphocyte infiltration into the joint, and degradation of cartilage when compared with control animals [15]. In addition, rats receiving *L casei *had lower levels of proinflammatory cytokines and reduced T cell proliferation, as well as increased production of the anti-inflammatory cytokine IL-10. Other studies have reported similar improvements in measures of arthritis after administration of *Lactobacillus fermentum *[16] and *Lactobacillus delbrueckii *[17].

A pilot clinical study [5] evaluated the long-term effects of LGG on symptoms of RA. In this double-blind study, 21 patients with RA were randomized to receive 2 capsules of LGG or placebo twice daily for 12 months. The mean number of tender and swollen joints decreased from 8.3 to 4.6 in the LGG group and from 5.5 to 4.8 in the placebo group (*P *= .41). However, considering only the long-term effects from baseline to 12 months, the mean number of tender and swollen joints decreased from 8.3 to 4.4 in the LGG group and increased from 5.5 to 5.6 in the placebo group (*P *= .09). RA activity was reduced in 71% of patients in the LGG group vs 30% of patients in the placebo group (*P *= .15). Although there were no statistical differences in clinical or biochemical parameters, more patients in the LGG group reported a greater feeling of well-being.

In the present study, patients with RA who received *Bacillus coagulans *GBI-30, 6086 experienced borderline statistically significant improvement from baseline in the Patient Pain Assessment score (*P *= .052) and statistically significant improvement from baseline in the Pain Scale (*P *= .046) vs placebo. Treatment with *Bacillus coagulans *GBI-30, 6086 resulted in greater improvement in patient global assessment, patient self-assessed disability, and reduction in total CRP. In addition, the ability to walk 2 miles, reach, and participate in daily activities steadily improved over the 60 days; however, these results did not reach statistical significance. The ability to arise, dress and groom, eat, grip, and maintain hygiene were similar between the 2 treatment groups. The overall results of this pilot study support a role for *Bacillus coagulans *GBI-30, 6086 as adjunctive therapy for inflammatory diseases, as well as affirm the pathophysiological connection between the gut microflora, the mucosal immune system, and arthritic diseases.

*Bacillus coagulans *GBI-30, 6086 is a gram-positive, spore-forming, aerobic to microaerophilic LAB bacillus [24] that has a demonstrated ability to improve gastrointestinal health. To be effective, probiotics must survive gastric and bile acids [25] in order to reach the intestinal tract, colonize the host epithelium, and exert a beneficial effect [26]. Most LAB probiotics are inactivated by bile and low gastric pH, whereas *Bacillus coagulans *GBI-30, 6086 cultures are protected by a hardened layer of organic spore coating that can withstand low gastric pH for delivery to the more favorable environment of the small intestine [20,25,27].

Although still controversial, evidence has suggested a possible causal link between gut microbes and systemic inflammatory disorders [5]. Once in the intestines, *Bacillus coagulans *GBI-30, 6086 is activated and releases anti-inflammatory molecules or acts indirectly to eradicate organisms in the gut responsible for the inflammatory immune response. Activated *Bacillus coagulans *GBI-30, 6086 produces bacteriocins [28] and lowers local pH by producing L(+) lactic acid that, along with competition for sites of mucosal adherence, works to dislodge and eliminate any antagonizing microbes that may be contributing to an inflammatory response. *Bacillus coagulans *GBI-30, 6086 also produces short-chain fatty acids such as butyric acid, a compound known to support the health and healing of cells in the small and large intestines and to contribute to modulation of the mucosal immune system.

While some RA studies follow subjects for a longer period of time, it was the feeling of the investigators that this study could be completed in a shorter timeframe. Many patients show relief from symptoms within 60 days when treated with other traditional anti-inflammatory agents such as NSAIDs, and the same would be expected of the study treatment. Therefore, subjects in this pilot study were treated and evaluated for 60 days.

## Conclusions

These study data suggest that *Bacillus coagulans *GBI-30, 6086 LAB probiotic may be a safe and effective adjunct therapy for the relief of symptoms of RA. Because of the low study population size, large-scale, controlled clinical trials are needed to confirm these results.

## Competing interests

This work was supported by a research grant from Ganeden Biotech, Inc. The investigators have no financial interest in the company.

## Authors' contributions

DM conceived of the study and participated in the study design, patient evaluations, and manuscript development. KE and JH participated in patient evaluations and coordination, and helped draft the manuscript. All authors have read and approved the final manuscript.

## Pre-publication history

The pre-publication history for this paper can be accessed here:

http://www.biomedcentral.com/1472-6882/10/1/prepub
